# Therapeutic potential of 5-aminolevulinic acid in metabolic disorders: Current insights and future directions

**DOI:** 10.1016/j.isci.2024.111477

**Published:** 2024-11-26

**Authors:** Olexandr Kuryata, Oleh Akimov, Mykola Riabushko, Heorhii Kostenko, Viktoriia Kostenko, Artur Mishchenko, Svetlana Nazarenko, Natalia Solovyova, Vitalii Kostenko

**Affiliations:** 1 Dnipro State Medical University, Department of Internal Medicine 2, Phthisiology, Occupational Diseases and Clinical Immunology, Dnipro, Ukraine; 2 Poltava State Medical University, Department of Pathophysiology, Poltava, Ukraine; 3 Poltava State Medical University, Department of Internal Medicine 2, Poltava, Ukraine; 4 Poltava State Medical University, Department of Foreign Languages with Latin and Medical Terminology, Poltava, Ukraine

**Keywords:** health sciences, medicine, medical specialty, therapeutics, natural sciences, biological sciences, physiology, endocrinology

## Abstract

5-Aminolevulinic acid (5-ALA) is an essential compound in the biosynthesis of heme, playing a critical role in various physiological processes within the human body. This review provides the thorough analysis of the latest research on the molecular mechanisms and potential therapeutic benefits of 5-ALA in managing metabolic disorders.

The ability of 5-ALA to influence immune response and inflammation, oxidative/nitrosative stress, antioxidant system, mitochondrial functions, as well as carbohydrate and lipid metabolism, is mediated by molecular mechanisms associated with the suppression of the transcription factor NF-κB signaling pathway, activation of the transcription factor Nrf2/heme oxygenase-1 (HO-1) system leading to the formation of heme-derived reaction products (carbon monoxide, ferrous iron, biliverdin, and bilirubin), which may contribute to HO-1-dependent cytoprotection through antioxidant and immunomodulatory effects. Additionally, it regulates the expression of peroxisome proliferator-activated receptor gamma coactivator 1-alpha, cytochrome *c* oxidase subunit IV, uncoupling proteins UCP1 and UCP2, glucose transporters GLUT1 and GLUT2, and sterol regulatory element-binding protein 1c in relevant tissues. Randomized controlled trials have confirmed the effects of 5-ALA on glucose control in both prediabetic and diabetic patients, noting its safety and tolerability, as well as the safety of its combined use with oral hypoglycemic agents. Only minor side effects have been reported. However, the impact of 5-ALA on markers of systemic inflammation, oxidative and nitrosative stress, and dyslipidemia was not evaluated in these studies. At the same time, preparations of 5-ALA may potentially be effective not only in the treatment of prediabetes and type 2 diabetes mellitus (T2DM), but also in other conditions associated with systemic inflammation, oxidative or nitrosative stress, mitochondrial dysfunction, as well as disorders of carbohydrate and lipid metabolism.

It has been concluded that the promising advancement of formulations containing 5-ALA may pave the way for new strategies in preventing and treating these diseases, with subsequent preclinical and clinical trials likely to follow.

## Graphical abstract

**Figure:**
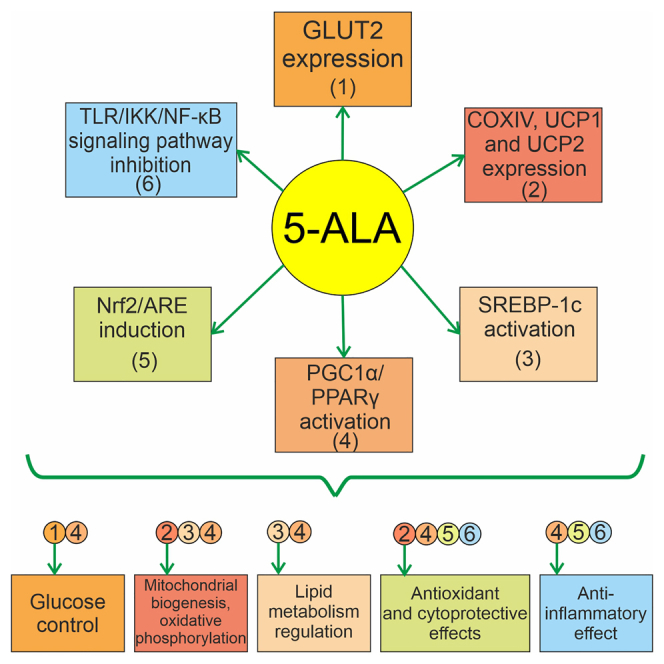

## Introduction

5-Aminolevulinic acid (5-ALA), also known as δ-aminolevulinic acid or 5-amino-4-oxopentanoic acid (IUPAC name), is an intriguing oxygen- and nitrogen-containing hydrocarbon that plays pivotal roles in biological systems. It serves as the universal precursor for a range of tetrapyrrole compounds, including essential molecules such as chlorophyll, heme, and vitamin B_12_.^1^^,^^2^ Synthesized across diverse kingdoms of life, including animals, plants, bacteria, and fungi, 5-ALA exhibits a versatile presence in nature. The abundance and ubiquity of 5-ALA are underscored by its endogenous nature, making it non-toxic to both humans and animals. Furthermore, its tendency to degrade easily in the environment, leaving behind no harmful residues, has garnered significant attention in recent scientific discourse.

There are two primary categories of 5-ALA applications in medical and healthcare settings. The first involves its use in combination with irradiation techniques, such as photoactivation, sonoactivation, or radioactivation, for treating malignant and inflammatory diseases. This approach leverages the accumulation of protoporphyrinogen IX, derived from 5-ALA, within the cytoplasm and mitochondria under conditions of anaerobic or aerobic glycolysis that are uncoupled with mitochondrial oxidative phosphorylation.^3^^,^^4^ Recent studies have collectively demonstrated the therapeutic efficacy of photodynamic therapy (PDT) with 5-ALA in treating a wide range of conditions, including keratosis, onychomycosis, lymphomas, meningiomas, malignant gliomas, basal cell carcinoma, Barrett’s esophagus, bladder cancer, brain tumors, gastric and other tumors, and in cosmetic procedures, shedding light on its potential as a cornerstone in medical interventions.^4^^,^^5^^,^^6^^,^^7^^,^^8^^,^^9^^,^^10^^,^^11^

Another approach to 5-ALA applications involves the production of heme in the presence of iron and its subsequent degradation by heme oxygenase (HO)-1.^12^^,^^13^ Recent studies have highlighted potential mechanisms through which 5-ALA may exert beneficial effects in metabolic disorders, particularly prediabetes and type 2 diabetes mellitus (T2DM),^14^^,^^15^^,^^16^^,^^17^^,^^18^^,^^19^ obesity,^20^ and atherosclerosis.^21^^,^^22^ These include its ability to mitigate chronic low-grade systemic inflammation and oxidative or nitrosative stress-induced damage, improve mitochondrial functions and insulin sensitivity, enhance glucose uptake in peripheral tissues, as well as decrease levels of circulating triglycerides, thus improving lipid profiles.

Additionally, 5-ALA is used as a farm animal feed additive to enhance blood iron levels, improve immune responses and disease resistance, stimulate growth, and promote egg production and quality, as well as milk composition.^23^^,^^24^^,^^25^^,^^26^^,^^27^ These studies highlight the potential benefits of supplementing with 5-ALA to enhance animal well-being and productivity, ultimately benefiting the welfare and economic sustainability of farming practices.

This review dives into the latest research on the molecular mechanisms and potential therapeutic benefits of 5-ALA in managing metabolic disorders.

A comprehensive search was conducted through electronic databases such as PubMed, Scopus, Web of Science, and the Cochrane Library to identify relevant literature sources. Traditional manual searching was also performed to determine relevant reference lists using key terms and phrases associated with the research question: “aminolevulinic acid”; “5-amino-4-oxopentanoic acid”; “aminolaevulinate”; “heme metabolism”; “5-aminolevulinic acid synthase”; “oxidative stress” or “nitrosative stress” or “immune response” or “inflammation” or “mitochondrial” or “transcription factors” or “pathogenesis” or “pathophysiology” AND “metabolic syndrome” or “diabetes mellitus” or “atherosclerosis” or “metabolic disorder,” etc. All experimental and clinical studies, alongside review articles, editorials, patent documentation, and case reports, were included without any restrictions on publication dates. Additionally, textbooks and other reference materials in the fields of biochemistry, molecular biology, pharmacology, pharmacy, endocrinology, clinical and molecular medicine were consulted. The vector graphics editor CorelDRAW 11 was used for creating the figures.

## Biochemical pathways of 5-aminolevulinic acid

The biochemical pathways governing the synthesis and metabolism of 5-aminolevulinic acid (5-ALA) are fundamental to understanding its role in cellular physiology and its potential therapeutic applications. As a crucial precursor in heme biosynthesis, 5-ALA occupies a central position in cellular metabolism, influencing various physiological processes.

### Enzymatic reactions in 5-aminolevulinic acid synthesis

**Figure 1:**
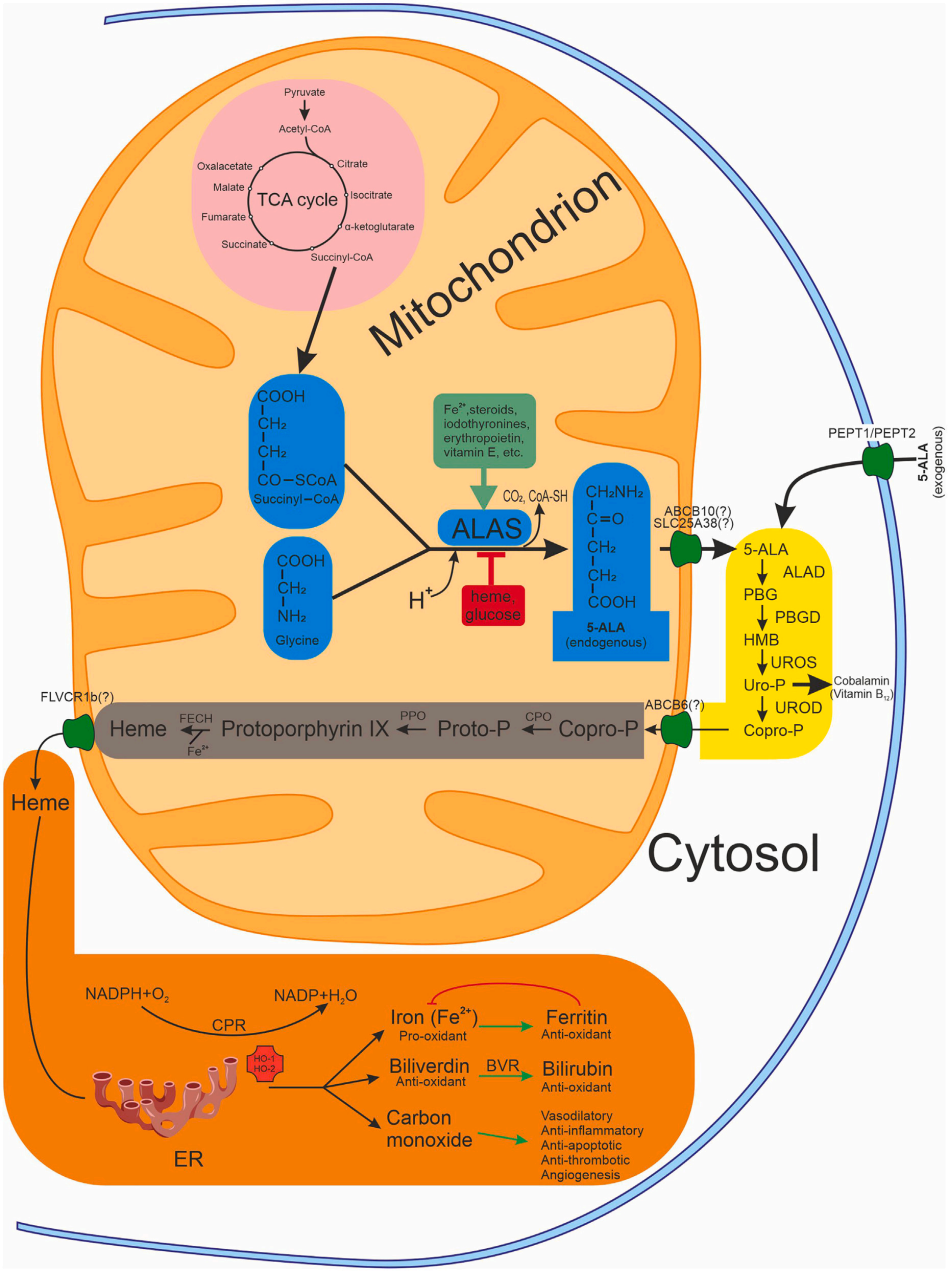
Biosynthetic reaction of the 5-ALA and its downstream pathways The figure is divided into three parts: central carbon metabolic pathway—TCA cycle (*pink*), 5-ALA synthetic pathway (*blue*), cytoplasmic and mitochondrial sections of downstream pathways (*yellow and gray*, respectively), and heme downstream metabolic pathway (*orange*). The *green* line indicates positive regulation, the *red* line indicates inhibition. Abbreviations: ABCB6, ATP-binding cassette subfamily B member 6; ABCB10, ATP-binding cassette subfamily B member 10; 5-ALA, 5-aminolevulinic acid; ALAD, 5-aminolevulinic acid dehydratase; ALAS, 5-aminolevulinic acid synthase; BVR, biliverdin reductase; CoA-SH, coenzyme A; Copro-P, coproporphyrinogen; CPO, coproporphyrinogen oxidase; CPR, cytochrome P450 reductase; ER, endoplasmaic reticulum; FECH, ferrochelatase; FLVCR1b, feline leukemia virus subgroup C cellular receptor 1b; HMB, hydroxymethylbiline; HO-1, heme oxygenase-1; HO-2, heme oxygenase-2; NADP, nicotinamide adenine dinucleotide phosphate; PBG, porphobilinogen; PBGD, porphobilinogen deaminase; PEPT1, peptide transporter 1; PEPT2, peptide transporter 2; PPO, protoporphyrinogen oxidase; Proto-P, protoporphyrinogen; SLC25A38, solute carrier family 25 member 38; TCA, tricarboxylic acid; UROD, uroporphyrinogen decarboxylase; Uro-P, uroporphyrinogen; UROS, uroporphyrinogen III synthase.

The 5-ALA synthesis is a critical process in cellular metabolism, serving as the precursor for heme, an essential molecule involved in oxygen transport, energy production, and numerous enzymatic reactions. The C4 (Shemin) pathway of 5-ALA biosynthesis (Figure 1) is characteristic not only of animals but also of yeast, some protozoa, and purple non-sulfur photosynthetic bacteria. Another pathway, known as the C5 pathway (also referred to as the Beale pathway), originated from the 5-ALA identification in *Chlorella vulgaris*.^1^ In the Shemin pathway, 5-aminolevulinic acid (5-ALA) is synthesized via an enzymatic reaction that occurs in the mitochondria. The synthesis of 5-ALA involves the condensation of glycine and succinyl-CoA, catalyzed by the enzyme 5-aminolevulinic acid synthase (ALAS), with pyridoxal 5′-phosphate serving as the co-factor.^28^ The production of succinyl-CoA is facilitated by methylmalonyl-CoA mutase, which relies on vitamin B_12_ as an indispensable co-factor. 5-ALA synthesis reaction represents a rate-limiting step in heme biosynthesis and is tightly regulated to maintain cellular heme homeostasis. ALAS exists in two isoforms: ALAS1, which is ubiquitously expressed and primarily responsible for heme synthesis in non-erythroid tissues, and ALAS2, which is predominantly expressed in erythroid cells.^29^ The activity of ALAS is regulated by various factors, including substrate availability, feedback inhibition by heme, and post-translational modifications, ensuring precise control over 5-ALA production.

### Regulation of 5-aminolevulinic acid synthesis

The synthesis of 5-ALA is subject to intricate regulatory mechanisms that ensure its production is finely tuned to cellular demands.^1^^,^^28^^,^^30^ Several factors influence the ALAS activity, including substrate availability, feedback inhibition by the end-product heme, and hormonal and metabolic signals (Figure 1). The ALAS expression is regulated at the transcriptional and post-transcriptional levels by various factors, including transcription factors, microRNAs, and epigenetic modifications. The synthesis of ALAS, which acts as a rate-limiting enzyme, is highly regulated through feedback mechanisms involving its *hemA* and *hemT* genes.^28^

The decrease in ALAS is associated with the ability of heme to suppress mRNA synthesis of the gene for this enzyme through the inhibition of RNA polymerase activity. It is recognized that glucose can impact heme metabolism. High glucose load has been observed to effectively treat acute attacks of inducible hepatic porphyria. This beneficial impact of glucose is believed to occur through the down-regulation of ALAS1 facilitated by the peroxisome proliferator-activated receptor γ coactivator 1α (PGC1α).^31^ The decrease in 5-ALA production may also result from an imbalance between the 5-ALA biosynthetic pathway and the tricarboxylic acid (TCA) cycle.^32^ Succinyl-CoA, a precursor of 5-ALA biosynthesis, acts as an intermediate in this cycle and plays a vital role in numerous metabolic processes.

Stimulators of ALAS induction include Fe^2+^ iron ions, steroid hormones (especially sex hormones), iodothyronines, vitamin E, and some drugs (barbiturates, estrogens, progestins, etc.).^1^^,^^28^^,^^30^^,^^33^^,^^34^ Most inducers affect ALAS synthesis similarly to lipophilic hormones (steroids), forming a ligand-receptor complex in the cytosol, which then translocates to the nucleus. Within the nucleus, the ligand-receptor complex acts as a transcriptional activator on the ALAS2 gene locus.

Physiological factors, such as blood oxygen saturation, also influence heme synthesis. Hypoxia-inducible factor-1-mediated ALAS2 upregulation has been demonstrated to enhance erythropoiesis, meeting the organism’s demands during conditions of low oxygen levels. This could be facilitated through elevated heme levels and an interaction between ALAS2 and erythropoietin.^35^

Numerous mechanisms regulate ALAS1 activity in response to heme levels: (1) transcriptional repression through a heme-responsive element; (2) post-transcriptional destabilization of ALAS1 mRNA; (3) post-translational inhibition via a heme regulatory motif; (4) direct inhibition of the activity of the enzyme, and (5) breakdown of ALAS1 protein via heme-mediated induction of the mitochondrial Lon peptidase 1.^29^

In erythroid cells, ALAS2 acts as a gatekeeper for producing large amounts of heme necessary for hemoglobin synthesis. Its synthesis rate transiently increases during active heme synthesis, regulated by the *trans*-activation of nuclear factor GATA1, CACC box, and NF-E2-binding sites in the promoter regions. ALAS2 mRNA translation is also controlled by the iron-responsive element/iron regulatory proteins binding system^36^ and microRNAs.^37^

The activity of ALAS is intricately regulated by post-translational modifications. For instance, the turnover of ALAS2 is controlled by the ubiquitin-proteasome system in the cytosol and by the caseinolytic mitochondrial matrix peptidase chaperone subunit X (CLPXP) in the mitochondria.^38^ Under normoxic conditions (21% O_2_), post-translational hydroxylation downregulates ALAS2 activity by promoting its ubiquitination and subsequent proteasomal degradation. The prolyl-4-hydroxylase/von Hippel-Lindau E3 ubiquitin ligase pathway is implicated in this process.^39^ Both ALAS proteins have mitochondrial targeting sequences that are believed to include heme-binding motifs. Two heme-binding motifs within the leader sequence, and one in the N-terminus of mature ALAS-1, are involved in heme-regulated translocation of ALAS1 *in vivo*.^40^ High heme concentrations promote its degradation by the CLPXP proteolytic complex and Lon protease within the mitochondria.^30^^,^^38^ Thus, post-translational modifications ensure precise regulation of ALAS activity, aligning heme biosynthesis with the cell’s physiological needs and stress responses.^41^

### Transmembrane transportation of endogenous and exogenous 5-ALA

Once synthesized in the mitochondrial matrix, 5-ALA is transported to the cytosol. While there are several candidate proteins believed to be crucial for 5-ALA transport, none have been directly confirmed *in vitro* to possess 5-ALA transport activity.^38^ Evidence suggests that the protein SLC25A38 (solute carrier family 25 member 38) may act as a transporter of glycine and 5-ALA across the mitochondrial inner membrane.^42^^,^^43^ This transport mechanism constitutes a critical and rate-determining step within the heme biosynthetic pathway.^43^ Previously, it was hypothesized that the protein ABCB10 is involved in the export of 5-ALA from the mitochondria to the cytosol. This hypothesis was supported by the observation that the addition of 5-ALA rescues defects observed in cardiomyocytes.^44^ However, a subsequent study has refuted the hypothesis that ABCB10 transports 5-ALA.^45^

5-ALA cellular influx transporters PEPT1 (SLC15A1) and PEPT2 (SLC15A2) belong to the solute carrier family 15 (SLC15), also known as the proton-coupled oligopeptide transporter (POT) family.^46^^,^^47^^,^^48^^,^^49^ PEPT1 and PEPT2 are known to be the most well-studied transporters responsible for the uptake, distribution and reabsorption of di- and tripeptides in the body.^49^ The intestinal brush border membrane primarily mediates the uptake of exogenous 5-ALA via PEPT1, while PEPT2 facilitates its transport in the kidney, mammary gland, brain, lung, and other organs.^46^^,^^47^^,^^48^ PEPT1 and PEPT2 function as secondary active transporters using an inward-directed electrochemical proton gradient as an energy source, which provides a driving force for the transport and accumulation of substances against a concentration gradient, resulting in intracellular concentrations higher than those in the extracellular space.^49^

Significantly, PEPT1-mediated peptide uptake has been demonstrated to attenuate inflammatory signaling pathways, including those involving nuclear factor kappa-light-chain-enhancer of activated B cells (NF-κB) and mitogen-activated protein kinases (MAPKs).^50^ This translates to a reduction in pro-inflammatory cytokine secretion and a decreased incidence of colitis in murine models.^51^

### Metabolism of 5-aminolevulinic acid

Following its synthesis, 5-ALA, in the presence of ferrous iron, undergoes further enzymatic transformations to produce protoporphyrin IX, the immediate precursor of heme. This process involves a series of enzymatic reactions within the heme biosynthetic pathway, occurring sequentially in the cytoplasm and mitochondria (Figure 1). In the cytosol, 5-ALA undergoes sequential conversions to generate porphobilinogen, hydroxymethylbilane, uroporphyrinogen III, and ultimately coproporphyrinogen III. Porphyrinogens are synthesized through the condensation polymerization of eight molecules of 5-ALA. Within the mitochondrion, coproporphyrinogen III is metabolized to protoporphyrinogen IX and then to protoporphyrin IX. Iron is subsequently inserted into protoporphyrin IX through a reaction catalyzed by ferrochelatase, leading to the formation of heme.^1^^,^^52^

Each enzymatic step is tightly regulated, ensuring the efficient production of heme while preventing the accumulation of toxic intermediates. Notably, disturbances in heme biosynthesis can lead to various disorders, such as porphyrias, underscoring the importance of maintaining proper regulation of 5-ALA metabolism.

### Heme downstream metabolic pathway

This pathway is essential for maintaining iron homeostasis and preventing the toxic accumulation of free heme (Figure 1), which can generate reactive oxygen species (ROS) and lead to cellular damage. Heme is transported out of mitochondria by FLVCR1b, a mitochondrial isoform of Feline leukemia virus subgroup C receptor 1 (FLVCR1). Overexpression of FLVCR1b stimulates heme synthesis and *in vitro* erythroid differentiation. Conversely, silencing FLVCR1b leads to mitochondrial heme accumulation and halted erythroid differentiation.^53^

The first and rate-limiting step in the heme degradation pathway is catalyzed by HO. This enzyme catalyzes the initial step in the oxidative breakdown of heme, converting it into ferrous iron (which is rapidly stored by ferritin), carbon monoxide (CO), and biliverdin-IXα, which is reduced to bilirubin-IXα by biliverdin reductase.^54^^,^^55^ There are two primary isoforms of heme oxygenase: HO-1 (inducible) and HO-2 (constitutive). HO-1 plays a particularly crucial role in cellular stress responses. Under conditions of oxidative stress, hypoxia, or inflammation, HO-1 expression is upregulated to protect cells from damage.^56^^,^^57^ HO-1 is strongly induced by a variety of stimuli, including its substrate heme, heat shock, heavy metal ions, ROS and reactive nitrogen species (RNS), inflammatory cytokines, and lipopolysaccharide (LPS).^55^

Oral administration of 5-ALA at 600 mg and sodium ferrous citrate (SFC) at 942 mg induced HO-1 expression in healthy human peripheral blood mononuclear cells at the 8-h time point. Neither 5-ALA nor SFC alone was able to induce HO-1 expression. Additionally, HO-1 was upregulated in blood myeloid and plasmacytoid dendritic cells following ALA+SFC treatment.^58^ Notably, HO-1 expression is regulated by transcription factors including NF-E2-related factor 2 (Nrf2), activator protein-1 (AP-1), and hypoxia-inducible factor, which integrate signaling pathway information at the HO-1 gene promoter.^59^

Recent decades have unveiled the beneficial properties of biliverdin, bilirubin, and biliverdin reductase A in a variety of biological processes, including their potent antioxidant capacities and immunomodulatory effects.^55^^,^^60^^,^^61^ CO, a gaseous signaling molecule, exhibits vasodilatory, anti-inflammatory, anti-apoptotic, anti-thrombotic, pro-angiogenic, and immunoregulatory properties.^13^^,^^54^^,^^62^^,^^63^ The levels of 5-ALA stored in the human body decline with age, resulting in diminished HO-1 expression within cells.

In summary, the heme downstream metabolic pathway, mediated by enzymes such as HO-1 and biliverdin reductase, plays a critical role in heme catabolism, protecting the body from heme-induced toxicity by degrading heme and generating biologically active molecules with significant physiological functions.

## Physiological and pharmacological functions of 5-aminolevulinic acid

The synthesis of 5-ALA plays a crucial role in cellular metabolism and physiology, serving as the starting point for heme biosynthesis. Heme, also known as iron protoporphyrin IX, serves as a prosthetic group in various hemoproteins, including respiratory cytochromes, gas sensors, P450 enzymes (CYPs), catalase, peroxidases, nitric oxide synthase (NOS), guanylyl cyclase, and even transcription factors.^52^^,^^53^^,^^54^^,^^55^^,^^56^^,^^57^^,^^58^^,^^59^^,^^60^^,^^61^^,^^62^^,^^63^^,^^64^^,^^65^ Heme also functions as a regulatory molecule in various cellular processes, including gene expression, circadian rhythm regulation, and response to oxidative or nitrosative stress.^66^ Conversely, hemin, the oxidized form of iron protoporphyrin IX, acts as a vital regulator of gene expression and promotes the growth of hematopoietic progenitor cells.^52^

### Effect of 5-aminolevulinic acid on immune response and inflammation

Only few clinical studies have investigated the beneficial effect of 5-ALA/SFC on biomarkers of systemic inflammatory response (SIR). Kaketani and Nakajima evaluated the efficacy of 5-ALA and SFC in treating COVID-19 patients with elevated C-reactive protein (CRP). Initial 5-ALA dosage was tailored to disease severity: 1,500 mg/day (500 mg thrice daily, approximately 25 mg/kg) for severe cases, and 750 mg/day (approximately 12.5 mg/kg) for mild cases.^67^

In an open-label, non-randomized pilot study, patients with moderate and severe COVID-19 received daily oral doses of 500 mg/750 mg 5-ALA and 286 mg/430 mg SFC, respectively, for the initial seven days. Subsequently, the dosage was reduced to 250 mg ALA and 143 mg SFC for the following 21 days in both groups. The severe group exhibited significantly lower levels of CRP, procalcitonin, and interleukin (IL)-6 compared to baseline. Notably, the severe group experienced a significantly shorter hospital stay (8 days) compared to the control group (16 days). Although these results are encouraging, larger-scale studies are needed to definitively establish the efficacy and safety of this treatment.^68^

However, most studies investigating the impact of 5-ALA on immune response and inflammation have been conducted in animal models and within the realm of veterinary medicine. Supplementation of 5-ALA in the diet of weaned castrated male pigs significantly increased levels of CD2^+^, CD8^+^, B cells, MHC-I, and MHC-II.^69^ 5-ALA enhances white blood cell counts and granulocytes, as well as the rate of phagocytosis and mitogen-induced proliferation of peripheral blood mononuclear cells in cows.^24^ Additionally, it has been demonstrated that 5-ALA might be beneficial as an immunomodulator, stimulating T cell activity through mild oxidative stress in growing broiler chickens, consequently enhancing growth performance.^23^

5-ALA has emerged as a potential therapeutic agent for modulating LPS-induced SIR due to its anti-inflammatory properties. Studies investigating the effects of 5-ALA supplementation on inflammatory markers have yielded mixed results. Some trials have reported decreases in pro-inflammatory cytokine levels, such as IL-2 and IL-6, interferon-γ, inducible NOS, and tumor necrosis factor (TNF)-like ligand 1A mRNA expression, following LPS injection.^23^ The plasma ceruloplasmin oxidase activity, a SIR marker, was reported to decrease in piglets following supplementation with 5-ALA.^70^ Furthermore, the ingestion of 5-ALA through the diet led to a reduction in plasma cortisol and TNF-α levels 2 h after LPS challenge.^71^ However, other studies have shown no significant changes in various inflammatory markers, including lymphocyte counts, haptoglobin levels, and Toll-like receptors (TLR) 2, 4, and 7 mRNA expression.^23^^,^^71^^,^^72^ Some conflicting findings have been reported regarding the relative weight of immune organs, such as the spleen, liver, bursa of Fabricius, and thymus gland.^23^^,^^73^^,^^74^

In summary, the literature suggests that the effects of 5-ALA supplementation on LPS-induced systemic inflammation vary and depend on various factors. While some studies indicate potential anti-inflammatory effects of 5-ALA, others report conflicting findings. Further research is needed to elucidate the mechanisms underlying the effects of 5-ALA on LPS-induced inflammation and to determine its therapeutic potential in inflammatory conditions.

Recent findings indicate that 5-ALA has emerged as a promising therapeutic approach for a range of conditions, including inflammatory diseases, autoimmune disorders, and transplantation. This potential is attributed to its anti-inflammatory, immunoregulatory and antioxidant properties, which are mediated through the induction of HO-1.^12^^,^^13^^,^^75^ Numerous studies have demonstrated the safety of typical doses of 5-ALA supplementation in both animals and humans, along with its anti-inflammatory properties.^13^^,^^68^^,^^76^

Recent studies by Chinese researchers have demonstrated that supplementing with 5-ALA aids liver regeneration by enhancing anti-inflammatory macrophage activity and promoting tissue repair. In mouse models of hepatic ischemia-reperfusion, 5-ALA enhanced liver metabolism and decreased inflammatory responses by shifting macrophages toward an anti-inflammatory M2 phenotype.^77^ Additionally, the suppression of the C-X3-C motif chemokine receptor 1 (CX3CR1) boosts this regenerative effect by increasing levels of insulin-like growth factor 1 and hepatocyte growth factor, further supporting liver recovery. The combined treatment with applying 5-ALA and CX3CR1 inhibition showed superior outcomes in promoting liver regeneration compared to 5-ALA alone, highlighting a potential therapeutic strategy for liver transplant and resection patients.^78^

The 5-ALA combined with SFC may alleviate ischemia-reperfusion injury in the mouse fatty liver model by reducing the expression of TLR 2 and 4, NF-κB, inflammatory cytokines, and ROS production in Kupffer cells.^79^ In an experiment involving LPS-challenged broiler chickens, dietary supplementation of 5-ALA significantly mitigated the increase in mRNA expression levels of hepatic TLR4, IL-1β, and IL-2.^27^ Another study demonstrated that the ability of 5-ALA to inhibit NF-κB activation was accompanied by a reduction in inducible NOS and cyclooxygenase-2 protein expression, subsequently leading to decreased production of nitric oxide and prostaglandin E2.^80^^,^^81^ The authors attribute this effect to the ability of 5-ALA/SFC to suppress LPS-induced phosphorylation of IκB kinase (IKK), degradation of the inhibitory protein IκBα, and the NF-κB subunit, thereby preventing further translocation of NF-κB into the nucleus.

These findings suggest that 5-ALA has significant potential as a therapeutic tool for managing inflammatory diseases and their complications by modulating key signaling pathways involved in SIR. Its capacity to induce HO-1 and inhibit NF-κB activation makes it a strong candidate for treating various inflammatory conditions.

### Effect of 5-aminolevulinic acid on oxidative/nitrosative stress and antioxidant system

Numerous studies have investigated the effects of 5-ALA on oxidative and nitrosative stress parameters in various experimental models.^25^^,^^27^ One research study observed a decrease in plasma ceruloplasmin oxidase activity, which typically elevates due to deficiency of antioxidant enzymes during iron deficiency, following 5-ALA administration (with or without iron).^70^ This suggests that 5-ALA may help regulate antioxidant activity, potentially mitigating the effects of iron deficiency. Another investigation found significantly lower levels of thiobarbituric acid reactive substances, lipid peroxidation products, in swine loin meat treated with a combination of 5-ALA and oriental medicinal plants compared to the control group.^82^ This implies that 5-ALA might aid in preserving tissues by mitigating oxidative damage.

A placebo-controlled, double-blind trial conducted on healthy volunteers revealed that supplementation with 5-ALA might enhance redox balance during high-intensity aerobic exercise.^83^ Specifically, the biological antioxidant potential and its ratio to diacron reactive oxygen metabolite showed significant differences in the onset of blood lactate accumulation state at week 4 in the group receiving 5-ALA compared to the placebo. A previous study conducted by Shinshu University in Japan demonstrated a decline in blood lactate concentrations among elderly female participants who took 5-ALA and SFC supplements.^84^^,^^85^

Some researchers suggest that 5-ALA, accumulated in acquired conditions like lead poisoning and inherited disorders such as intermittent acute porphyria, may serve as contributing sources of oxyradicals and oxidative stress in these diseases.^86^ However, having thoroughly examined various literature sources, Hendawy et al.^25^ concluded that the antioxidant properties of 5-ALA may be linked to its ability to induce a mild level of oxidative stress, leading to cellular preconditioning against ROS/RNS. Specifically, ROS/RNS induction supports the generation of antioxidants by activating Nrf2, enhances autophagy, and strengthens intracellular defenses against pathogens. LPS treatment is known to elevate ROS and RNS production, while the production of HO-1 increases following exposure to various stress stimuli. Moreover, in addition to their antioxidant properties, both HO-1 and Nrf2 inhibit oxidative/nitrosative stress and SIR by suppressing NF-κB.^87^^,^^88^^,^^89^^,^^90^^,^^91^^,^^92^

Given its antioxidant properties, 5-ALA holds promise as a potential therapeutic approach for preventing and managing a range of oxidative stress-related conditions, such as cardiovascular diseases, neurodegenerative disorders, metabolic syndrome, and T2DM.

### Effect of 5-aminolevulinic acid on mitochondrial functions

5-ALA is an important precursor of heme, essential for the activities of mitochondrial respiratory complexes II, III, and IV, as well as cytochrome *c*. It exerts an influence on mitochondrial energy metabolism by enhancing adenosine triphosphate (ATP) production and promoting mitochondrial respiration. It has also been revealed that the heme synthetic enzyme ALAS, also known as Hem1 in yeast, along with guanosine triphosphatases (GTPases) that control mitochondrial dynamics machinery *(Mgm1* and *Dnm1)* and endoplasmic reticulum contact sites (*Gem1*), play a role in regulating the flow of heme between the mitochondria and nucleus.^93^

Several studies have documented an elevation in ATP levels post-5-ALA treatment, indicating its ability to stimulate mitochondrial energy generation. Recently, it has been demonstrated that administering a combination of 5-ALA hydrochloride and SFC augments the activities of complexes II and IV, boosts ATP production, and alleviates defective phenotypes associated with complexes I deficiency in *Drosophila*.^94^

In another study, the effects of different concentrations of 5-ALA on fibroblasts from 8 individuals with mitochondrial diseases and healthy controls were investigated. Results showed that in normal fibroblasts, 5-ALA upregulated the expression levels of oxidative phosphorylation complex subunits and corresponding genes.^95^ Moreover, treatment with 5-ALA led to increased oxygen consumption rate and ATP levels in normal fibroblasts, as well as enhanced the levels of HO-1 protein and mRNA in all fibroblasts, and increased the relative mitochondrial DNA (mtDNA) copy number. These findings suggest that 5-ALA effectively enhances oxidative phosphorylation, HO-1 protein, and mtDNA.

It is noteworthy that the use of 5-ALA is considered a novel approach to anti-atherosclerotic therapy. Previous research identified mitochondrial mutations linked to atherosclerosis, leading to the hypothesis that targeting mitochondria burdened with these mutations could be beneficial. As a result, 5-ALA is being considered as a promising agent for this purpose.^22^ By targeting key aspects of mitochondrial biology, 5-ALA holds promise as a therapeutic agent for mitigating mitochondrial dysfunction-associated disorders, including metabolic diseases.

### Effect of 5-aminolevulinic acid on carbohydrate and lipid metabolism

5-ALA has garnered significant attention in recent years due to its potential effects on carbohydrate metabolism. Research indicates that 5-ALA may play a role in modulating key pathways involved in glucose utilization and homeostasis. Studies have demonstrated that 5-ALA supplementation can improve glucose tolerance and insulin sensitivity in various animal models and human subjects. The data provides *in vivo* evidence indicating that 5-ALA deficiency diminishes mitochondrial function and leads to impaired glucose tolerance and insulin resistance (IR) in an age-dependent manner.^96^ Muscle mitochondrial activation in mice treated with 5-ALA is suggested to be beneficial for treating sarcopenia and glucose intolerance.^97^ In addition, supplementation with 5-ALA induces beneficial alterations in lipid metabolism, leading to decreased levels of circulating triglycerides and enhancements in lipid profiles.^98^

Recent studies have demonstrated that oral administration of 5-ALA may offer protection against mild hyperglycemia and serve as a preventive measure against T2DM.^14^^,^^15^^,^^16^^,^^17^^,^^18^^,^^19^ These investigations strongly suggest an association between 5-ALA or heme and glucose metabolism *in vivo*. In a study conducted on Zucker diabetic fatty rats, it was observed that the oral administration of 5-ALA in combination with SFC for 6 weeks led to a reduction in plasma glucose and hemoglobin A1c (HbA1c) levels, without impacting plasma insulin levels.^99^ Additionally, the 5-ALA/SFC treatment significantly improved glucose tolerance. Notably, the administration of 5-ALA/SFC induced the HO-1 expression in white adipose tissue and liver, and the levels of induced HO-1 expression correlated with the glucose-lowering effects of 5-ALA/SFC. These findings suggest that 5-ALA combined with ferrous iron effectively reduces hyperglycemia T2DM without affecting plasma insulin levels. The induction of HO-1 may play a role in the mechanisms underlying the glucose-lowering effect of 5-ALA/SFC.

Furthermore, heme has been shown to regulate hepatic glucose production by modulating the expression of key enzymes involved in gluconeogenesis and glycogenolysis. Animal studies have demonstrated that both 5-ALA and heme have the potential to decrease hepatic glucose output and enhance liver insulin sensitivity, thereby playing a role in maintaining overall glucose homeostasis. In hepatic cells, the transcriptional repressor Rev-erbα detects heme via its heme-binding domain, essential for its repressor function.^100^ This detection mechanism plays a crucial role in regulating glucose homeostasis by inhibiting glucose production and the expression of gluconeogenic genes such as glucose 6-phosphatase and phosphoenolpyruvate carboxykinase. Moreover, Rev-erbα controls circadian rhythms by regulating the Bmal1 gene and modulates energy metabolism by supplying heme for mitochondrial respiration, among other functions.^101^

There is growing evidence indicating that the combination of 5-ALA and SFC may have positive effects on the function and survival of pancreatic β-cells by enhancing mitochondrial function within these cells. Indeed, numerous studies have demonstrated the advantageous impact of 5-ALA/SFC on glycemic control among individuals with prediabetes and T2DM.^14^^,^^15^^,^^16^^,^^17^^,^^18^^,^^19^

The research conducted on obese Wistar rats fed a high-fat diet revealed that administering 5-ALA/SFC in different dosages daily for 6 months effectively reduced plasma glucose levels.^102^ The study showed a significant reduction in the Homeostasis Model Assessment of Insulin Resistance (HOMA-IR) index in the groups treated with 5-ALA/SFC, suggesting its effectiveness in improving IR. However, it also suggested that 5-ALA/SFC might have no impact on pancreatic β cells. Despite the enhancement of appetite by 5-ALA/SFC, reductions in both body weight and visceral fat were noted. The authors suppose that the promotion of appetite might rely on the regulation of two appetite-regulating factors, namely amylin and peptide YY. Additionally, adiponectin, monocyte chemotactic protein-1, and TNF-α may contribute to glucose and lipid metabolism. It is important to note that 5-ALA/SFC likely regulated the expression of cytochrome *c* oxidase subunit IV (COXIV), uncoupling protein 1 and 2 (UCP1 and UCP2), glucose transporter 2 (GLUT2), and sterol regulatory element-binding protein 1c (SREBP-1c) in relevant tissues. This regulation helps maintain homeostasis in mitochondrial metabolism, decrease accumulation of fat in the liver, reduce visceral fat deposition, and further enhance glucose and fat metabolism. These findings suggest that 5-ALA/SFC could ameliorate hyperglycemia and dyslipidemia, decrease fat accumulation and body weight by promoting mitochondrial activity, and modulating genes related to lipid and glucose metabolism.

5-ALA administration induced exogenous HO-1 production at plaque sites in low-density lipoprotein (LDL) receptor-deficient mice, leading to improved lipid profiles (including reduced oxidized LDL) and attenuated atherosclerotic plaque progression *in vivo*.^21^ Another investigation affirms that 5-ALA/SFC effectively decreased body weight, fat mass, hepatic lipid accumulation, and enhanced blood glucose levels and oral glucose tolerance test outcomes in diabetic mice subjected to a high-fat diet for 9 weeks.^20^ Furthermore, these substances inhibited the augmented glomerular tuft area, correlated with elevated HO-1 protein expression. Researchers concluded that 5-ALA/SFC shows promise in addressing conditions associated with obesity or T2DM, such as diabetic nephropathy and nonalcoholic fatty liver disease. It was found that 5-ALA activates the AMP-activated protein kinase (AMPK) signaling pathway, leading to increased lipolysis and fatty acid β-oxidation.^103^ Treatment of human hepatocarcinoma (HepG2) cells with 5-ALA resulted in elevated expression of lipolysis-related genes, including PGC-1α. These findings suggest that 5-ALA may have potential as a novel treatment for nonalcoholic fatty liver disease by restoring AMPK phosphorylation and acetyl-CoA levels, thereby enhancing the expression of PGC1α and carnitine palmitoyltransferase 1α.

These findings underscore the promise of 5-ALA as a therapeutic agent for preventing and managing T2DM and other metabolic disorders characterized by impaired carbohydrate and lipid metabolism.

## Therapeutic applications of 5-aminolevulinic acid and its prospects as a treatment for metabolic disorders

The medical applications of 5-ALA extend beyond its primary role in heme synthesis, encompassing diverse therapeutic potentials. One notable application of 5-ALA is in PDT, utilized for treating various cancers, skin conditions, and cosmetic imperfections.^4^^,^^5^^,^^104^^,^^105^ In PDT, exogenously administered 5-ALA is preferentially taken up by tumor cells and metabolized to protoporphyrin IX, which upon activation by light leads to the generation of cytotoxic ROS, resulting in tumor cell death. Recently, the potential application of 5-ALA as a photosensitizer in photodynamic therapy for the comprehensive treatment of chronic wounds in patients with ischemic and mixed forms of diabetic foot syndrome has been identified.^10^ Combined light exposure using the photosensitizer not only stimulates the ROS formation but also enhances the effect of phagocytic cells. This enhancement is evident in the activation of neutrophil chemotaxis, adhesion, and endocytosis. The PDT utilizing 5-ALA as a photosensitizer augments the molecular mechanisms involved in intercellular interaction at all stages of primary immune system activation.

Additionally, 5-ALA has shown promise in the management of neurological disorders, such as Alzheimer’s disease, through its neuroprotective and anti-inflammatory effects.^106^^,^^107^ Findings suggest that 5-ALA and SFC may be promising therapeutic agents for combating SARS-CoV-2 infection by suppressing viral growth and mitigating the risk of post-COVID conditions.^108^^,^^109^^,^^110^ Moreover, emerging research suggests potential therapeutic avenues for 5-ALA in addressing prediabetes and T2DM,^18^^,^^20^^,^^76^^,^^111^^,^^112^^,^^113^ obesity,^20^ and cardiovascular diseases.^22^^,^^114^^,^^115^^,^^116^^,^^117^

**Figure 2:**
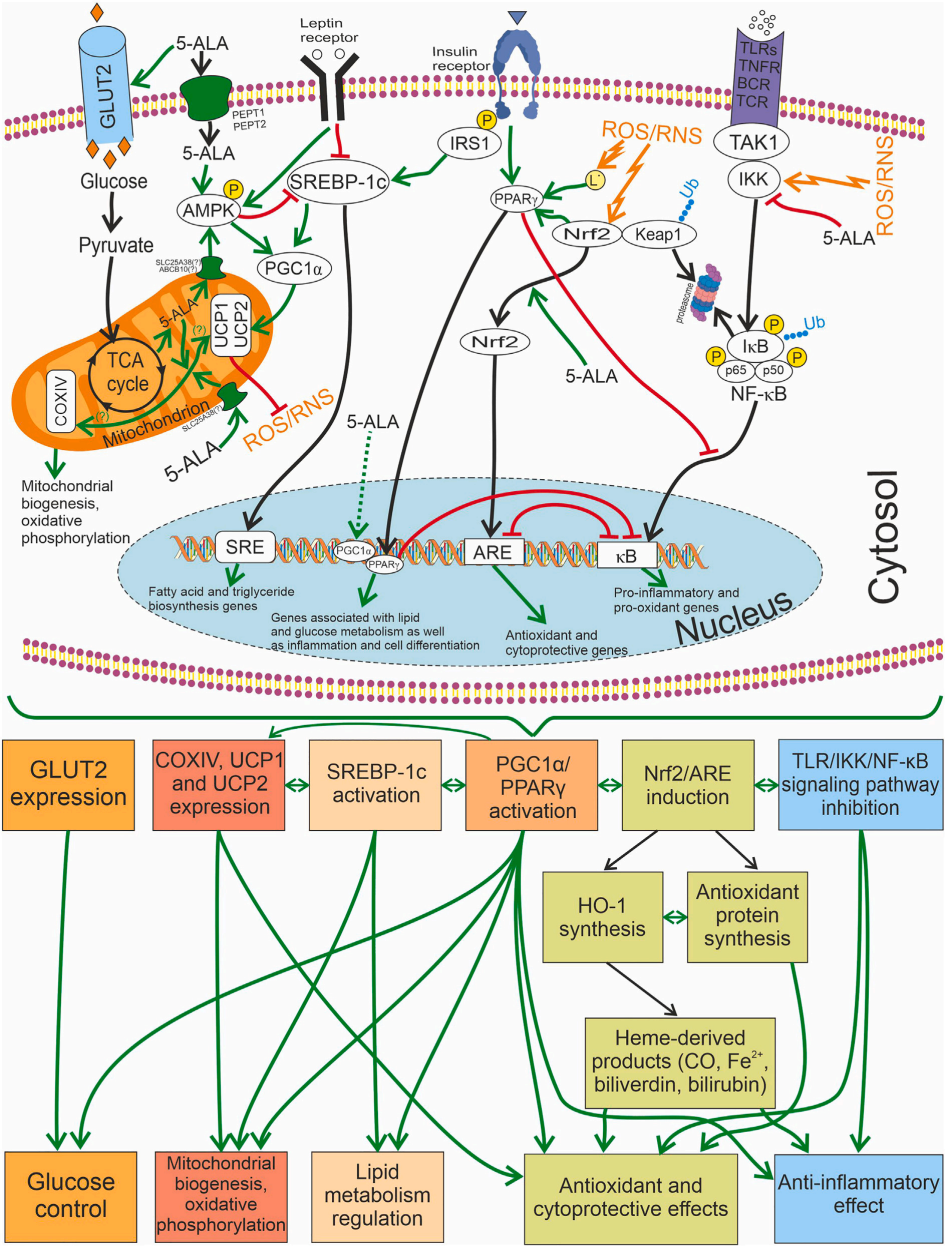
The molecular mechanisms underlying the physiological and pharmacological effects of 5-ALA (in the presence of Fe^2+^) The *green* line indicates positive regulation, the *red* line indicates inhibition, the *dashed* line represents indirect effects. 5-ALA reveals an anti-inflammatory effect by inhibiting the NF-κB signaling pathway through IKK and activating PPARγ signaling (via PGC1α). Its impact on pro-oxidant cascades is mitigated by its ability to induce the Nrf2/ARE signaling pathway and PPARγ. Enhanced Nrf2-dependent synthesis of HO-1 leads to the production of heme-derived reaction products (CO, ferrous iron, biliverdin, and bilirubin), which may contribute to cytoprotection through antioxidant and immunomodulatory effects. Additionally, 5-ALA, whether imported into the mitochondrion exogenously (possibly via transport proteins like SLC25A38) or endogenously produced, regulates the expression of mitochondrial proteins (COXIV, UCP1, and UCP2), promoting mitochondrial biogenesis and activating oxidative phosphorylation. The increase in SREBP-1c expression driven by 5-ALA enhances the biosynthesis of fatty acids and triglycerides; however, it is subject to inhibitory control by another target of 5-ALA, AMPK. Furthermore, AMPK activates UCP1 and UCP2 via PGC1α. The decrease in mitochondrial membrane potential (ΔΨ) due to UCP2 may reduce the potential for ROS/RNS formation in mitochondria: this occurs by decreasing the efficiency of electron transport, which reduces the likelihood of free radical formation. By increasing the expression of GLUT1 and GLUT2, 5-ALA enhances the cellular uptake of glucose. Once inside the cell, glucose can undergo glycolysis, a metabolic process that generates ATP. This promotes glucose uptake and utilization, supporting the energy requirements of the cell, thus ensuring proper cellular function and metabolism. Abbreviations: ABCB10, ATP-binding cassette subfamily B member 10; 5-ALA, 5-aminolevulinic acid; AMPK, adenosine monophosphate-activated protein kinase; ARE, antioxidant response element; BCR, B cell receptor; COXIV, cytochrome *c* oxidase subunit IV; GLUT1, glucose transporter 1; GLUT2, glucose transporter 2; HO-1, heme oxygenase-1; IκB, NF-κB inhibitory protein; IKK, IκB kinase; IRS-1, insulin receptor substrate 1; Keap1, Kelch-like ECH-associated protein 1; L**˙**, lipid radical; NF-κB, nuclear factor κB; Nrf2, nuclear factor erythroid 2-related factor 2; P, phosphorylation; PEPT1, peptide transporter 1; PEPT2, peptide transporter 2; PGC1α, peroxisome proliferator-activated receptor γ coactivator 1α; PPARγ, peroxisome proliferator-activated receptor γ; ROS, reactive oxygen species; RNS, reactive nitrogen species; SLC25A38, solute carrier family 25 member 38; SRE, sterol regulatory element; SREBP-1c, sterol regulatory element-binding protein 1c; TAK1, transforming growth factor-β-activated kinase; TCA, tricarboxylic acid; TCR, T cell receptor; TLRs, Toll-like receptors; TNFR, tumor necrosis factor receptor; Ub, ubiquitination; UCP1, uncoupling protein 1; UCP 2, uncoupling protein 2.

Current research supports the involvement of SIR, mitochondrial dysfunction, the production of damaging ROS/RNS, as well as decreased insulin secretion and increased IR in metabolic syndrome^118^^,^^119^^,^^120^^,^^121^^,^^122^ and T2DM.^123^^,^^124^ Examining the mechanism of action of 5-ALA, an amino acid synthesized in the mitochondria, and a novel supplement containing 5-ALA that has recently appeared on the market, research demonstrates its impact on diminishing pro-inflammatory and pro-oxidant TLR/NF-κB-dependent signaling pathways. Moreover, it activates anti-inflammatory signaling systems such as Nrf2-antioxidant responsive element, and induces HO-1, which breaks down heme into CO, biliverdin-IXα, and bilirubin-IXα, known to provide cytoprotective effects. The molecular mechanisms underlying the physiological and pharmacological effects of 5-ALA in SIR and metabolic disorders are schematically depicted in Figure 2.

The effectiveness of 5-ALA/SFC application in prediabetes and T2DM, including those treated with oral hypoglycemic agents, is confirmed by clinical and experimental studies (Table 1).

**Table 1:** Efficacy of 5-ALA/SFC in treatment of metabolic disorders and its mechanisms

Metabolic disorder	Mechanism of action of 5-ALA	Reference
T2DM conditions in Otsuka Long-Evans Tokushima Fatty rats	5-ALA ameliorates diabetic abnormalities in rats by reducing visceral fat mass (in the retroperitoneal region) through a decrease in adipocyte mitochondrial content	Sato et al.^111^
Lipid accumulation in hepatocytes and hyperglycemia during obesity and T2DM	5-ALA administration with ferrous citrate increases aerobic glycolysis, lipolysis and leads to higher expression of HO-1 and UCP1	Kamiya et al.^20^
Muscle and mitochondrial abnormalities during T2DM	5-ALA administration with ferrous citrate changes skeletal muscle transcriptome leading to increased expression of glucose uptake and mitochondrial oxidative phosphorylation-related genes	Kitamura et al.^112^
Decreased glucose tolerance during high-fat diet	5-ALA administration with ferrous citrate increases GLUT1 translocation in the plasma membrane without affecting GLUT1 expression	Kuroda et al.^113^
Increased fasting blood glucose level and decreased glucose tolerance in the patients with mitochondrial diabetes mellitus	5-ALA administration with ferrous citrate reduces blood glucose levels and improves glucose tolerance by enhancing mitochondrial function	Nakamura et al.^18^
Increased intensity of anaerobic glycolysis during breast cancer	5-ALA administration leads to increased heme synthesis, which destabilizes Bach1 and decreases anaerobic glycolysis in favor of TCA cycle activation through AMPK	Kaur et al.^125^
Endoplasmic reticulum stress caused by excessive intake of palmitic acid	5-ALA induces HO-1 expression and decreases Bach1 expression	Hamada et al.^126^

Clinical studies have demonstrated that the use of 5-ALA offers the benefit of minimal adverse effects, as its safety and tolerance have been established in clinical trials.^14^^,^^15^^,^^16^^,^^17^^,^^18^^,^^19^^,^^127^^,^^128^ Additionally, it is expected to be more cost-effective compared to certain oral hypoglycemic agents.^16^^,^^17^ However, further research is required to confirm the efficacy of 5-ALA in diabetes treatment, particularly in terms of reducing HbA1c levels. Concerning safety, 5-ALA has been avoided in patients with porphyria due to the pathophysiology of the disease. Generally, studies involving 5-ALA have consistently reported low levels of adverse events, with some studies even indicating no adverse events at all. Reported events such as common cold symptoms, menstrual pain, diarrhea, and headache were typically mild, with no serious adverse events documented.^76^

Researchers have also shown significant enhancements in self-perception of effort expenditure, reduced feelings of loneliness, and improved coping abilities among middle-aged and older adults with prediabetes after a 12-week regimen of 5-ALA supplementation.^129^ These improvements in mood and coping skills may help individuals overcome emotional barriers that hinder the adoption of healthy lifestyle practices, thereby aiding in the prevention of diabetes onset. These findings are consistent with the results of a randomized, double-blind, placebo-controlled, parallel study demonstrating oral 5-ALA reduces weakness and negative mood in individuals experiencing persistent physical fatigue.^130^

## Conclusions

Beyond its primary function in heme synthesis, 5-ALA demonstrates a range of physiological effects, serving as a signaling molecule that influences cellular processes including oxidative stress response, mitochondrial function, and gene expression. Moreover, recent research highlights its involvement in cellular metabolism, indicating potential implications for energy regulation and the management of metabolic disorders.

The ability of 5-ALA to influence immune response and inflammation, oxidative/nitrosative stress, antioxidant system, mitochondrial functions, as well as carbohydrate and lipid metabolism is mediated by molecular mechanisms associated with the suppression of the TLR/IKK/NF-κB signaling pathway, activation of the Nrf2/HO-1 system leading to the formation of heme-derived reaction products (CO, ferrous iron, biliverdin, and bilirubin), which may contribute to HO-1-dependent cytoprotection through antioxidant and immunomodulatory effects. Additionally, it regulates the expression of PGC1α, COXIV, UCP1, UCP2, GLUT1, GLUT2, and SREBP-1c in relevant tissues.

The majority of studies investigating the physiological or pharmacological effects of 5-ALA have been conducted on farm animals. The findings obtained from these studies require further preclinical and clinical validation. An important aspect of further research is to clarify the significance of combining 5-ALA with ferrous iron preparations.

Randomized controlled trials have confirmed the effects of 5-ALA on glucose control in both prediabetic and diabetic patients, noting its safety and tolerability, as well as the safety of its combined use with oral hypoglycemic agents. Only minor side effects have been reported. However, the impact of 5-ALA on markers of systemic inflammation, oxidative and nitrosative stress, and dyslipidemia was not evaluated in these studies.

At the same time, preparations of 5-ALA may potentially be effective not only in the treatment of prediabetes and T2DM, but also in other conditions associated with systemic inflammation, oxidative or nitrosative stress, mitochondrial dysfunction, as well as disorders of carbohydrate and lipid metabolism.

New strategies for preventing and treating these diseases may depend on the promising development of formulations containing 5-ALA, followed by subsequent preclinical and clinical trials.

## Declaration of interests

Authors declare no competing interests.
